# Mitochondrial genomes and Doubly Uniparental Inheritance: new insights from *Musculista senhousia *sex-linked mitochondrial DNAs (Bivalvia Mytilidae)

**DOI:** 10.1186/1471-2164-12-442

**Published:** 2011-09-06

**Authors:** Marco Passamonti, Andrea Ricci, Liliana Milani, Fabrizio Ghiselli

**Affiliations:** 1 Department of Biologia Evoluzionistica Sperimentale, University of Bologna, Bologna, Italy

## Abstract

**Background:**

Doubly Uniparental Inheritance (DUI) is a fascinating exception to matrilinear inheritance of mitochondrial DNA (mtDNA). Species with DUI are characterized by two distinct mtDNAs that are inherited either through females (F-mtDNA) or through males (M-mtDNA). DUI sex-linked mitochondrial genomes share several unusual features, such as additional protein coding genes and unusual gene duplications/structures, which have been related to the functionality of DUI. Recently, new evidence for DUI was found in the mytilid bivalve *Musculista senhousia*. This paper describes the complete sex-linked mitochondrial genomes of this species.

**Results:**

Our analysis highlights that both M and F mtDNAs share roughly the same gene content and order, but with some remarkable differences. The *Musculista *sex-linked mtDNAs have differently organized putative control regions (CR), which include repeats and palindromic motifs, thought to provide sites for DNA-binding proteins involved in the transcriptional machinery. Moreover, in male mtDNA, two *cox2 *genes were found, one (M-*cox2b*) 123bp longer.

**Conclusions:**

The complete mtDNA genome characterization of DUI bivalves is the first step to unravel the complex genetic signals allowing Doubly Uniparental Inheritance, and the evolutionary implications of such an unusual transmission route in mitochondrial genome evolution in Bivalvia. The observed redundancy of the palindromic motifs in *Musculista *M-mtDNA may have a role on the process by which sperm mtDNA becomes dominant or exclusive of the male germline of DUI species. Moreover, the duplicated M-COX2b gene may have a different, still unknown, function related to DUI, in accordance to what has been already proposed for other DUI species in which a similar *cox2 *extension has been hypothesized to be a tag for male mitochondria.

## Background

Metazoan mitochondrial DNA (mtDNA) is generally a small molecule (15-20 kb), and although much larger mitochondrial genomes have occasionally been found, they are often products of duplications of mtDNA portions, rather than variations in gene content [1,2]. The typical mitochondrial gene complement encodes 13 protein subunits of the oxidative phosphorylation enzymes, 2 rRNAs and 22 tRNAs. However, the coding sequences (CDS) can be up to 16, the tRNAs up to 27 (source MitoZoa: http://mi.caspur.it/mitozoa see [3]), and the rRNAs can be duplicated and/or fragmented in discontinuous genes, as in oysters [4]. Generally, there is also a single large non-coding region that is known to contain regulatory elements for replication and transcription (i.e. 'Control Region', CR), but it is unclear whether it is homologous among distantly related animals or, alternatively, it independently arose from various non-coding sequences. This difficulty in establishing homology is because CRs share sequence similarity only among closely related taxa. Finally, the mtDNA is almost always a circular molecule: only the cnidarian classes Cubozoa, Scyphozoa and Hydrozoa have been found to have linear mtDNA chromosomes [5]. All metazoan mitochondrial genes have homologs in plants, fungi and/or protists [6-9].

The Mollusca is the second largest animal Phylum and currently 99 complete mitochondrial genomes are available in Genbank; among those, only 38 are from Bivalvia, the second class in terms of species richness among mollusks. So far, bivalve mtDNA displays an extraordinary amount of variation in gene arrangement, i.e. very few shared gene boundaries are detectable, and gene translocations are common across all gene classes (protein-coding genes, tRNAs and rRNAs). For this reason, bivalve mitochondrial genome may provide an excellent experimental system to review and test models of mt gene rearrangement evolution, which were mainly developed in groups with stable genomes, such as vertebrates or arthropods. In addition, gene duplications and/or losses are present in almost every bivalve taxon in which a complete mitochondrial genome is available (see [10]). It is therefore evident that efforts should be made to improve the knowledge of bivalve mitochondrial genomes.

Another interesting feature of bivalve mtDNA is its unusual transmission route, which is found in some species: while in Metazoa mtDNA is known to be usually transmitted by Strict Maternal Inheritance (SMI; [11,12]), some bivalve mollusks show a deviation from this rule, named Doubly Uniparental Inheritance (DUI; [13,14]). DUI was found in species belonging to seven different bivalve families: Donacidae, Hyriidae, Margaritiferidae, Mytilidae, Solenidae, Unionidae, and Veneridae ([15,16]). Species with DUI are characterized by the presence of two distinct gender-associated mtDNAs: one transmitted through eggs (F) and one transmitted through sperm (M). The F and M genomes show up to 52% nucleotide divergence [17]. DUI seems at first to violate the universal rule of uniparental inheritance of organelles, because males receive their mtDNA from both parents and their tissues are heteroplasmic. However the two mtDNAs segregate independently: the F-type is transmitted to the next generation only through females, while the M-type is only transmitted from father to sons, therefore both genomes are actually transmitted uniparentally.

Because of its unique features, DUI should be a choice model to address many aspects of a wide range of biological sub-fields such as mitochondria inheritance, mtDNA evolution and recombination, genomic conflicts, evolution of sex and developmental biology (see [18] for a review).

Recently, evidence for a new example of DUI was found in the mytilid *Musculista senhousia *[19]. In this work we characterized the two sex-linked mitochondrial genomes of *M. senhousia*, a step forward to the complete genetic characterization of DUI related sex-linked mitochondrial genomes. In fact, several unusual features are coming to light when analyzing mtDNAs in DUI systems, such as additional protein coding genes ([20], and references therein) and gene duplications/features [21,22]. Functional explanations for these features will require much additional work, but are needed to understand the evolution and maintenance of DUI.

## Results

### Mitochondrial genome features in *M. senhousia*

The obtained *M. senhousia *mtDNAs are 21,557 bp long in female (F-type) and 20,612 bp in male (M-type) (see Tables 1 and 2). Sequences are available in GenBank (Acc. No. GU001953-GU001954). The size of both F and M mitochondrial genomes are within the size range of mollusk mtDNAs sequenced to date, i.e. from 7808 bp in *Batilaria cumingi *to 32,115 bp in *Placopecten magellanicus *(source MitoZoa: http://mi.caspur.it/mitozoa; [3]).

**Table 1:** Organization of female *Musculista senhousia *mitochondrial genome.

Type	Name	Starts	Stops	Length	Strand	Anticodon	Start Codon	Stop Codon
GENE	nad3	1	390	390	H		ATG	TAA
UR	UR-1	391	625	235				
tRNA	trnY	626	691	66	H	GTA		
UR	UR-2	692	1234	543				
tRNA	trnH	1235	1299	65	H	GTG		
UR	UR-3	1300	1315	16				
tRNA	trnI	1316	1381	66	H	GAT		
UR	UR-4	1382	1391	10				
tRNA	trnN	1392	1457	66	H	GTT		
UR	UR-5	1458	1564	107				
tRNA	trnE	1565	1631	67	H	TTC		
LUR	LUR	1632	6152	4521				
GENE	cox1	6153	7736	1584	H		ATG	TAA
UR	UR-6	7737	8114	378				
GENE	cox2	8115	8774	660	H		ATA	TAA
UR	UR-7	8775	8832	58				
GENE	atp8	8833	8967	135	H		ATG	TAA
UR	UR-8	8968	9051	84	H			
GENE	atp6	9052	9765	714	H		ATG	TAG
UR	UR-9	9766	9791	26				
tRNA	trnT	9792	9858	67	H	TGT		
GENE	cob	9835	11031	1197	H		ATA	TAA
UR	UR-10	11032	11049	18				
tRNA	trnD	11050	11114	65	H	GTC		
UR	UR-11	11115	11123	9				
tRNA	trnR	11124	11189	66	H	TCG		
tRNA	trnS(AGN)	11191	11248	58	H	TCT		
UR	UR-12	11249	11268	20				
tRNA	trnG	11269	11336	68	H	TCC		
rRNA	rrnS	11337	12154	818	H			
GENE	nad6	12155	12778	624	H		ATG	TAA
UR	UR-13	12779	12828	50				
GENE	nad2	12829	13773	945	H		ATA	TAA
UR	UR-14	13774	13855	82				
GENE	cox3	13856	14710	855	H		ATG	TAA
UR	UR-15	14711	14721	11				
tRNA	trnK	14722	14792	71	H	TTT		
UR	UR-16	14793	14797	5				
tRNA	trnF	14798	14865	68	H	GAA		
UR	UR-17	14866	14878	13				
tRNA	trnP	14879	14945	67	H	TGG		
UR	UR-18	14946	14977	32				
tRNA	trnL(CUN)	14978	15042	65	H	TAG		
UR	UR-19	15043	15047	5				
tRNA	trnC	15048	15114	67	H	GCA		
UR	UR-20	15115	15159	45				
tRNA	trnL(UUR)	15160	15223	64	H	TAA		
UR	UR-21	15224	15259	36				
GENE	nad1	15260	16252	993	H		ATG	TAA
UR	UR-22	16253	16385	133				
tRNA	trnM(AUA)	16386	16448	63	H	TAT		
UR	UR-23	16449	16486	38				
tRNA	trnV	16487	16550	64	H	TAC		
UR	UR-24	16551	16695	145				
GENE	nad4L	16696	16911	216	H		ATA	TAA
UR	UR-25	16912	16988	77				
GENE	nad5	16989	18738	1750	H		ATA	T--
tRNA	trnA	18739	18804	66	H	TGC		
UR	UR-26	18805	18843	39				
GENE	nad4	18844	20163	1320	H		ATA	TAG
UR	UR-27	20164	20213	50				
tRNA	trnW	20214	20280	67	H	TCA		
UR	UR-28	20281	20285	5				
tRNA	trnQ	20286	20353	68	H	TTG		
UR	UR-29	20354	20360	7				
tRNA	trnM(AUG)	20361	20427	67	H	CAT		
rRNA	rrnL	20428	21557	1130	H			

**Table 2:** Organization of male *Musculista senhousia *mitochondrial genome.

Type	Name	Starts	Stops	Length	Strand	Anticodon	Start Codon	Stop Codon
GENE	nad3	1	375	375	H		ATG	TAA
UR	UR-1	376	433	58				
tRNA	trnY	434	501	68	H	GTA		
UR	UR-2	502	533	32				
tRNA	trnH	534	599	66	H	GTG		
UR	UR-3	600	618	19				
tRNA	trnI	619	688	70	H	GAT		
tRNA	trnN	687	753	67	H	GTT		
LUR	LUR	754	3597	2844				
tRNA	trnE	3598	3668	71	H	TTC		
UR	UR-4	3669	3708	40				
GENE	cox1	3709	5292	1584	H		ATG	TAA
UR	UR-5	5293	5852	560				
GENE	cox2b	5853	6665	813	H		ATG	TAA
UR	UR-6	6666	6706	41				
GENE	cox2	6707	7396	690	H		ATA	TAA
UR	UR-7	7397	7402	6				
GENE	atp8	7403	7594	192	H		ATG	TAG
UR	UR-8	7595	7612	18				
GENE	atp6	7613	8326	714	H		ATG	TAA
UR	UR-9	8327	8347	21				
tRNA	trnT	8348	8416	69	H	TGT		
GENE	cob	8392	9588	1197	H		ATA	TAA
UR	UR-10	9589	9606	18				
tRNA	trnD	9607	9671	65	H	GTC		
UR	UR-11	9672	9681	10				
tRNA	trnR	9682	9745	64	H	TCG		
tRNA	trnS(AGN)	9747	9806	60	H	TCT		
UR	UR-12	9807	9825	19				
tRNA	trnG	9826	9893	68	H	TCC		
rRNA	rrnS	9894	10793	900	H			
GENE	nad6	10794	11417	624	H		ATG	TAA
UR	UR-13	11418	11472	55				
GENE	nad2	11473	12417	945	H		ATA	TAG
UR	UR-14	12418	12444	27				
GENE	cox3	12445	13299	855	H		ATG	TAG
tRNA	trnK	13299	13366	68	H	TTT		
UR	UR-15	13367	13377	11				
tRNA	trnF	13378	13445	68	H	GAA		
UR	UR-16	13446	13464	19				
tRNA	trnP	13465	13528	64	H	TGG		
UR	UR-17	13529	13554	26				
tRNA	trnL(CUN)	13555	13621	67	H	TAG		
UR	UR-18	13622	13625	4				
tRNA	trnC	13626	13696	71	H	GCA		
UR	UR-19	13697	13737	41				
tRNA	trnL(UUR)	13738	13804	67	H	TAA		
UR	UR-20	13805	13840	36				
GENE	nad1	13841	14836	996	H		ATG	TAG
tRNA	trnM(AUA)	14835	14899	65	H	TAT		
UR	UR-21	14900	14985	86				
tRNA	trnV	14986	15049	64	H	TAC		
UR	UR-22	15050	15183	134				
GENE	nad4L	15184	15399	216	H		ATA	TAA
UR	UR-23	15400	15464	65				
GENE	nad5	15465	17229	1765	H		ATA	T--
tRNA	trnA	17230	17294	65	H	TGC		
UR	UR-24	17295	17338	44				
GENE	nad4	17339	18667	1329	H		ATA	TAA
UR	UR-25	18668	18710	43				
tRNA	trnW	18711	18777	67	H	TCA		
UR	UR-26	18778	18781	4				
tRNA	trnQ	18782	18848	67	H	TTG		
UR	UR-27	18849	18863	15				
tRNA	trnM(AUG)	18864	18930	67	H	CAT		
rRNA	rrnL	18931	20612	1682	H			

**Figure 1:**
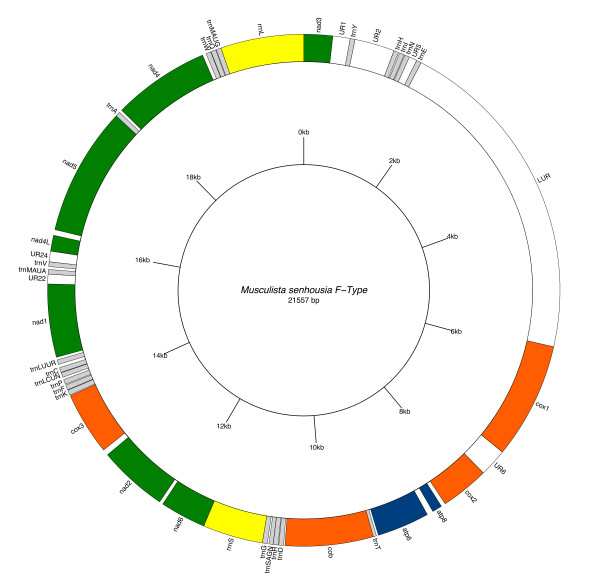
**Female *Musculista senhousia *mitochondrial genome**. Gene map of the female *Musculista senhousia *mitochondrial genome. Shortest URs (< 100 bp) are not indicated.

**Figure 2:**
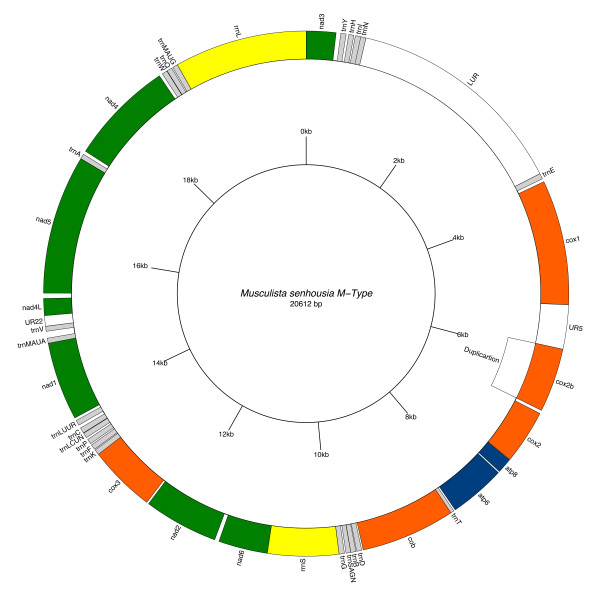
**Male *Musculista senhousia *mitochondrial genome**. Gene map of the male *Musculista senhousia *mitochondrial genome. Shortest URs (< 100 bp) are not indicated.

*M. senhousia *F and M gene arrangements are remarkably different from other fully sequenced metazoan mtDNAs (see [10] for a review). Genome annotations are reported in Figure 1 and 2, Table 1 and 2. When compared to other Mytilidae, only four gene boundaries are shared with *Mytilus *(tRNAs are not considered), i.e. *rrnS-nad6, nad2-cox3, nad4L-nad5 *and *nad3-cox1*, while the rest of the genome is different, thus highlighting that gene arrangement evolves rapidly within the family.

Comparing the two sex linked genomes, protein-coding genes may have different lengths (Table 3). Both F-type and M-type include a large number of Unassigned Regions (URs; 29 in F and 27 in M: see Tables 1, 2 and Additional File 1). Among these, the largest (4,521 and 2,844 bp in female and male respectively) are here referred as LURs (i.e. Large Unassigned Regions).

**Table 3:** Length, base composition and sequence divergence of M, F genes and URs in *Musculista senhousia*.

Gene/Region	F/M	Length	Base Composition (% T, C, A, G)				pD ± SE
UR1-27/LUR	M	4296	37.8	11.2	31.4	19.5	NA
UR1-29/LUR	F	6798	37.9	10.4	30.8	20.9	NA
*rrnL*	M	1682	37.3	12.6	30.8	19.3	0.343 ± 0.015
	F	1130	35.8	13.5	30.4	20.3	
*rrnS*	M	900	36.0	11.6	33.1	19.3	0.093 ± 0.009
	F	818	37.2	11.0	32.2	19.7	
all rRNA genes	M	2582	36.3	12.2	31.6	19.3	0.209 ± 0.010
	F	1948	36.4	12.4	31.2	20.0	
*atp6*	M	714	43.8	12.7	23.5	19.9	0.258 ± 0.016
	F	714	42.2	12.9	23.8	21.1	
*atp8*	M	192	42.2	14.1	27.6	16.1	0.281 ± 0.037
	F	135	43.0	12.6	25.9	18.5	
*cox1*	M	1584	38.3	15.9	24.7	21.1	0.180 ± 0.009
	F	1584	40.0	14.4	24.4	21.3	
*cox2*	M	690	36.7	15.2	26.7	21.4	0.264 ± 0.016
	F	660	37.4	14.5	27.3	20.8	
*cox2b*	M	813	35.9	14.1	28.7	21.3	0.267 ± 0.016*
	F	NA	NA				
*cox3*	M	855	42.0	13.1	23.3	21.6	0.220 ± 0.012
	F	855	43.4	12.9	20.9	22.8	
*cob*	M	1197	40.6	13.9	25.2	20.3	0.106 ± 0.009
	F	1197	40.4	13.6	24.9	21.1	
*nad1*	M	996	39.8	12.2	26.0	22.0	0.227 ± 0.014
	F	993	41.3	11.5	24.4	23.2	
*nad2*	M	945	44.9	10.8	24.4	19.9	0.302 ± 0.013
	F	945	44.1	10.9	22.4	22.5	
*nad3*	M	375	44.3	14.1	21.3	20.3	0.267 ± 0.021
	F	390	45.6	12.6	21.0	20.8	
*nad4*	M	1329	41.4	11.5	23.6	23.5	0.273 ± 0.013
	F	1320	39.9	11.9	24.3	23.9	
*nad4L*	M	216	43.5	8.8	24.5	23.1	0.199 ± 0.027
	F	216	44.0	8.8	24.5	22.7	
*nad5*	M	1765	39.5	13.2	27.9	19.4	0.285 ± 0.011
	F	1750	38.7	13.3	25.7	22.3	
*nad6*	M	624	43.8	11.4	25.6	19.2	0.217 ± 0.017
	F	624	42.1	12.3	25.2	20.4	
all proteins	M	12295	40.6	13.2	25.4	20.9	0.231 ± 0.004#
	F	11383	40.9	12.8	24.1	22.1	
complete	M	20612	39.3	12.7	27.7	20.3	NA
	F	21557	39.3	12.0	27.2	21.4	

UR = Unassigned Regions.

NA = Not Available.

pD = p-Distance.

SE = Standard Error.

*: pD between *Mcox2 *and *Mcox2b *genes.

#: *Mcox2b *gene was excluded from the computation of overall pD.

Both F and M mt genomes show the same gene order and contain the full gene complement of the typical metazoan mtDNA, with two additional tRNAs: *trnM *and *trnL *(Figures 1 and 2; Tables 1 and 2). In males the *cox2 *gene is duplicated (Figure 2 and Table 2).

The *atp8 *gene was reported as missing in several bivalve mollusks, however, as recently reported [23], the lack of *atp8 *would rather be an annotation inaccuracy due to the extreme variability of the gene. Following [23], we found an *atp8 *gene in *M. senhousia *in both M and F genomes.

The position of the two ribosomal RNA genes, obtained through BLAST comparison, does not differ between male and female. In both sexes, *rrnL *is located in a region flanked by the *trnM(AUG) *and *nad3 *genes. Assuming that the first base at the 5'-end comes immediately after the *trnM(AUG)*, and the 3'-end of the gene corresponds to the first base upstream of the start codon of *nad3 *gene, the length of the *rrnL *genes are remarkably different: the male *rrnL *(1,682 bp in length) is 552 bp longer than the female one (1,130 bp in length). The *rrnS *gene is located in a region flanked by *trnS *and *nad6 *genes and, as above, we assumed that the first base at the 5'-end comes immediately after *trnG*, and that the 3'-end of the gene corresponds to the first base upstream of the start codon of *nad6 *gene. Here, the difference in length is reduced to 82 bp: the female *rrnS *gene is 819 bp long while the male one is 1,087 bp.

F and M genomes of *M. senhousia *contain 22 tRNA genes (see Tables 1, 2 and Additional File 2). As observed in mtDNA of some other mollusks (*Katharina tunicata*, *Cepaea nemoralis*, *Mytilus *species complex and *Argopecten irradians*), two leucine tRNA genes are present in *M. senhousia*. These can be differentiated by their anticodons: TAA for *trnL(UUR) *and TAG for *trnL(CUN)*, which are 2-fold and 4-fold redundant respectively. Consequently, *tnrL *is 6-fold redundant. An additional *trnM *was also detected, as in *V. philippinarum*, *Mytilus *species complex, *Crassostrea gigas*, *C. hongkongensis *and *C. virginica*. The additional tRNA coding for methionine, *trnM(AUA)*, has the TAT anticodon.

In both male and female mtDNAs, *trnS(AGN) *have a shortened DHU (See Additional File 2) that is not atypical, as this arm is unpaired in many metazoan taxa [24-27]. Moreover, mispairing between bases in stems is consistent across several taxa. For example, the second base pair in the anticodon stem of *trnW *has a T-T mispairing in *Lampsilis ornata*, *Mytilus*, and *K. tunicata *and a T-G pairing in several gastropods [25].

**Figure 3:**
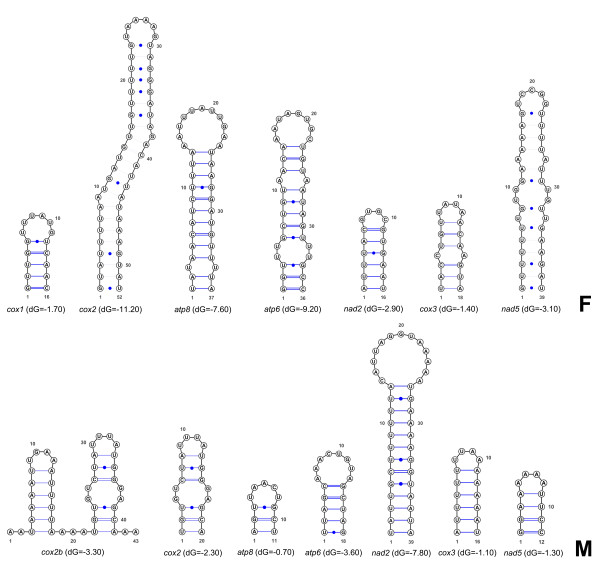
**Intergenic palindromes**. Putative secondary structures preceding the 5'-end of some protein-coding genes. (F) Female *Musculista senhousia *mitochondrial genome. (M) Male *Musculista senhousia *mitochondrial genome.

In the F mitochondrial genome of *Musculista*, 20 out of 22 tRNA genes are clustered in five groups of two to six (see Figure 1 and Table 1). Of the remaining two, *trnT *lies between *atp6 *and the 5'-end of *cob *genes (with 24 bp overlapping each other) while *trnA *lies between *nad5 *and *nad4 *genes. Thus, 4 of the 13 protein-coding genes (*cob*, *nad1*, *nad4L *and *nad4*) have a tRNA preceding their 5'-end. In contrast, 7 other genes (*cox1*, *cox2*, *atp8*, *atp6*, *nad2*, *cox3 *and *nad5*) have a non-coding sequence at their 5'-end that is capable of forming a stem and loop structure (see Figure 3).

In male mitochondrial DNA, 19 of the 22 tRNA genes are clustered in five groups ranging from two to six (see Figure 2 and Table 2). Of the remaining three, *trnT *lies between *atp6 *and the 5'-end of *cob *genes (with 25 bp overlapping each other), *trnA *lies between *nad5 *and *nad4 *genes and *trnE *lies between the large unassigned region (LUR) and the 5'-end of *cox1 *gene. Thus, 5 of the 14 protein-coding genes (*cox1*, *cob*, *nad1*, *nad4L *and *nad4*) have a tRNA preceding their 5'-end, while 7 other genes (*cox2b*, *cox2, atp8, atp6*, *nad2*, *cox3 *and *nad5*) have a non-coding sequence preceding their 5'-end that is capable of forming a stem and loop structure (see Figure 3). In a few cases those structures contain the translation initiation codon (*cox1 *and *cox2 *in females, *nad2 *in males).

The nucleotide compositions of the two genomes are summarized in Table 3. Given the G content of the F and M coding strand (see Table 3), this can be considered as the heavy (H) strand of the molecule. The A+T content of the H strand is also high (66.5%, F; 67.0%, M). Variable values of A+T content are common in mollusks, and they have been reported in *L. ornata *(62%, [28]), *Pupa strigosa *(61.1%, [29]), and *C. nemoralis *(59.8%, [25]). In other mollusks, the A+T content is much higher *(Albinaria coerulea*, 70.7%, [30]; *K. tunicata*, 69.0%, [6]; *Graptame eborea*, 74.1%, [31]). *Musculista *values in A+T content are among the highest observed in the Phylum, and reflect the high heterogeneity of molluscan mtDNA [2]. Moreover, there is a marked bias in favor of T against C, which is not restricted to any particular class of genes and does not differ between the two genomes.

The GC and AT asymmetry between the two mitochondrial DNA strands can be expressed in terms of GC skew and AT skew calculated according to [32]: GC skew = (G-C)/(G+C) and AT skew = (A-T)/(A+T), where G, C, A, and T are the occurrences of the four bases in the H strand. In *M. senhousia *F and M mitochondrial genomes, the GC skew and the AT skew are F: +0.28 and -0.18, and M: +0.23 and -0.17, respectively.

In the *M. senhousia *male mtDNA 6 out of 14 protein genes start with the ATA codon and 8 with ATG, while in the female 7 out of 13 start with ATG and 6 with ATA (Tables 1 and 2). This pattern differs from that observed for *Mytilus galloprovincialis*, where 9 out of 13 protein genes start with the ATG codon, 2 with the ATA and 2 with GTG [23,33]. In all known metazoan mtDNAs, the most common start codon is ATG, and it is a general opinion that the methionine tRNA with the CAT anticodon represents the ancestral form. Moreover [24] suggested that the second methionine tRNA arose by duplication. The F and M genomes of the venerid *Venerupis philippinarum *also have two tRNA genes for methionine, but both have the ancestral CAT anticodon. TAA is the termination codon ten times in F and nine times in M mtDNA, while TAG is a stop codon two times in F, and four times in M. In both M and F genomes, *nad5 *gene is terminated by an incomplete termination codon T-- (Tables 1 and 2), with their likely completion occurring by polyadenylation after transcript processing [34].

A total of 4,098 and 3,794 amino acids residues are encoded by male and female *M. senhousia *mitochondrial genome respectively (Table 4). All codons do occur in both *Musculista *mitochondrial genomes (Table 5). UUU (phenylalanine) is the most frequent codon, followed by UUA (leucine). UUU is also the most frequent codon in *M. galloprovincialis *[33], in *L. ornata *[28] and in *C. nemoralis *[35], whereas UUA (leucine) is most common in *A. coerulea *[30], *P. strigosa *[29], *Roboastra europaea *[36], *G. eborea *[31], and *K. tunicata *[6]. These two codons are also the most frequently used in other invertebrate mtDNAs [37-42]. UUU is also very frequent in basal chordates (e.g. amphioxus, *Branchiostoma lanceolatum*, [43]), but not in most vertebrates, where CUA (e.g., *Cyprinus*, [44]; *Homo sapiens*, [45]) or AUU (e.g., *Xenopus laevis*, [46]; *Danio rerio*, [47]) are the most frequent.

**Table 4:** Genes, gene lengths and divergences in male and female *Musculista senhousia *protein coding genes.

Protein gene	**M**^**aa**^	**F**^**aa**^	pD ± SE	Ks	Ka	Ka/Ks
*atp6*	238	238	0.228 ± 0.026	0.894	0.156	0.17
*atp8*	64	45	0.302 ± 0.070	0.581	0.233	0.40
*cox1*	528	528	0.053 ± 0.009	0.838	0.042	0.05
*cox2*	230	220	0.251 ± 0.027	0.877	0.178	0.20
cox2b*	271	NA	0.279 ± 0.029*	0.653*	0.223*	0.35*
*cox3*	285	285	0.155 ± 0.022	0.811	0.107	0.13
*cob*	399	399	0.058 ± 0.012	0.346	0.034	0.10
*nad1*	332	331	0.218 ± 0.022	0.670	0.145	0.22
*nad2*	315	315	0.306 ± 0.026	0.843	0.244	0.29
*nad3*	125	130	0.218 ± 0.034	0.964	0.162	0.17
*nad4*	443	440	0.243 ± 0.020	0.931	0.175	0.19
*nad4L*	72	72	0.183 ± 0.045	0.626	0.107	0.17
*nad5*	588	583	0.274 ± 0.018	0.862	0.208	0.24
*nad6*	208	208	0.324 ± 0.031	0.619	0.268	0.43
all proteins	4,098	3,794		0.716	0.143	0.20

M^aa ^and F^aa ^= number of amino acids in male and female respectively.

pD = p-Distances at the amino acidic level.

Ks and Ka = divergence of protein genes in synonymous (Ks) and non synonymous (Ka) sites respectively.

SE = Standard Error.

Ka/Ks = ratio values between Ka and Ks.

*: comparisons between *Mcox2 *and *Mcox2b *genes.

**Table 5:** Codon usage in male and female *Musculista senhousia *mitochondrial genomes.

FEMALE																		
**aa**	**Codon**	**Count**	**%**	**aa**	**Codon**	**Count**	**%**	**aa**	**Codon**	**Count**	**%**	**aa**	**Codon**	**Count**	**%**	**Codon**	**Count**	%

Phe (F)	UUU	303	8,0	Ser (S)	UCU	107	2,8	Tyr (Y)	UAU	125	3,3	Cys (C)	UGU	80	2,1	UNU	615	16,2
	**UUC**	36	0,9		UCC	8	0,2		**UAC**	39	1,0		**UGC**	14	0,4	UNC	97	2,6
Leu (L)	**UUA**	254	6,7		UCA	36	0,9	s.c. (*)	UAA	14	0,4	Trp (W)	**UGA**	53	1,4	UNA	357	9,4
	UUG	105	2,8		UCG	14	0,4		UAG	7	0,2		UGG	50	1,3	UNG	176	4,6
	CUU	89	2,3	Pro (P)	CCU	95	2,5	His (H)	CAU	58	1,5	Arg (R)	CGU	35	0,9	CNU	277	7,3
	CUC	20	0,5		CCC	13	0,3		**CAC**	15	0,4		CGC	7	0,2	CNC	55	1,5
	**CUA**	62	1,6		**CCA**	11	0,3	Gln (Q)	**CAA**	32	0,8		**CGA**	14	0,4	CNA	119	3,1
	CUG	41	1,1		CCG	4	0,1		CAG	26	0,7		CGG	13	0,3	CNG	84	2,2
Ile (I)	AUU	147	3,9	Thr (T)	ACU	54	1,4	Asn (N)	AAU	82	2,2	Ser (S)	AGU	71	1,9	ANU	354	9,3
	**AUC**	41	1,1		ACC	9	0,2		**AAC**	27	0,7		AGC	30	0,8	ANC	107	2,8
Met (M)	**AUA**	139	3,7		**ACA**	29	0,8	Lys (K)	**AAA**	81	2,1		**AGA**	90	2,4	ANA	339	8,9
	**AUG**	62	1,6		ACG	17	0,4		AAG	33	0,9		AGG	68	1,8	ANG	180	4,7
Val (V)	GUU	200	5,3	Ala (A)	GCU	88	2,3	Asp (D)	GAU	59	1,6	Gly (G)	GGU	102	2,7	GNU	449	11,8
	GUC	24	0,6		GCC	17	0,4		**GAC**	15	0,4		GGC	39	1,0	GNC	95	2,5
	**GUA**	113	3,0		**GCA**	44	1,2	Glu (E)	**GAA**	44	1,2		**GGA**	43	1,1	GNA	244	6,4
	GUG	84	2,2		GCG	22	0,6		GAG	49	1,3		GGG	89	2,3	GNG	244	6,4

	NUN	1720	45,4		NCN	568	15,0		NAN	706	18,6		NGN	798	21,0	Total	3792	

**MALE**																		

**aa**	Codon	Count	%	**aa**	Codon	Count	%	**aa**	Codon	Count	%	**aa**	Codon	Count	%	Codon	Count	%

Phe (F)	UUU	333	8,1	Ser (S)	UCU	131	3,2	Tyr (Y)	UAU	133	3,2	Cys (C)	UGU	90	2,2	UNU	687	16,8
	**UUC**	57	1,4		UCC	22	0,5		**UAC**	36	0,9		**UGC**	15	0,4	UNC	130	3,2
Leu (L)	**UUA**	274	6,7		UCA	36	0,9	s.c. (*)	UAA	18	0,4	Trp (W)	**UGA**	69	1,7	UNA	397	9,7
	UUG	104	2,5		UCG	6	0,1		UAG	10	0,2		UGG	46	1,1	UNG	166	4,1
	CUU	86	2,1	Pro (P)	CCU	91	2,2	His (H)	CAU	51	1,2	Arg (R)	CGU	42	1,0	CNU	270	6,6
	CUC	16	0,4		CCC	14	0,3		**CAC**	30	0,7		CGC	11	0,3	CNC	71	1,7
	**CUA**	55	1,3		**CCA**	20	0,5	Gln (Q)	**CAA**	40	1,0		**CGA**	12	0,3	CNA	127	3,1
	CUG	28	0,7		CCG	8	0,2		CAG	22	0,5		CGG	8	0,2	CNG	66	1,6
Ile (I)	AUU	178	4,3	Thr (T)	ACU	61	1,5	Asn (N)	AAU	81	2,0	Ser (S)	AGU	78	1,9	ANU	398	9,7
	**AUC**	43	1,0		ACC	22	0,5		**AAC**	52	1,3		AGC	43	1,0	ANC	160	3,9
Met (M)	**AUA**	148	3,6		**ACA**	35	0,9	Lys (K)	**AAA**	104	2,5		**AGA**	97	2,4	ANA	384	9,4
	**AUG**	79	1,9		ACG	12	0,3		AAG	38	0,9		AGG	75	1,8	ANG	204	5,0
Val (V)	GUU	193	4,7	Ala (A)	GCU	81	2,0	Asp (D)	GAU	65	1,6	Gly (G)	GGU	103	2,5	GNU	442	10,8
	GUC	30	0,7		GCC	22	0,5		**GAC**	22	0,5		GGC	28	0,7	GNC	102	2,5
	**GUA**	106	2,6		**GCA**	44	1,1	Glu (E)	**GAA**	59	1,4		**GGA**	53	1,3	GNA	262	6,4
	GUG	83	2,0		GCG	19	0,5		GAG	42	1,0		GGG	88	2,1	GNG	232	5,7

	NUN	1813	44,2		NCN	624	15,2		NAN	803	19,6		NGN	858	20,9	Total	4098	

Codons that match the corresponding tRNA anticodon are bold and underlined.

aa: coded amminoacid.

s.c.: stop codon.

The least used codons in males are UCG (6), CCG (8) and CGG (8), while in females they are CCG (4), CGC (7) and UAG (7). Of these, CGC is also among the least common in the mtDNA of other mollusks. Synonymous codons, whether four-fold (4FD) or two-fold (2FD) degenerate, are recognized by the same tRNA, with the exception of the methionine codons, which are recognized by different tRNAs (Table 5).

Moreover, 2,754 F and 2,967 M *Musculista *codons (72.6% and 72.4% in female and in male respectively) end with an A or T, a more pronounced phenomenon than what observed for a typical invertebrate codon bias. There is a strong bias against the use of C (9.3% and 11.3% in female and in male respectively) at the third position nucleotide in all codons: in detail, for residues with a fourfold degenerate third position, codon families ending with T are the most frequently used (46.7% and 46.6% in female and male respectively). This is also the case for two-fold degenerate codons. In other words, in every case an amino acid residue can be specified by any NNY codon, both female and male *M. senhousia *mitochondrial genomes have a much higher proportion of NNT:NNC. In fact, female showed 44.7% of T and 9.3% of C, with NNT:NNC ratio of 4.8:1; while in male the ratio's value is slightly lower: 3.9:1 (43.8% of T and 11.2% of C). At the second position, there is even a stronger bias in favor of the use of T usage (45.4% and 44.2% in female and male respectively)(see Table 6), like in *M. edulis *(43.5%), *C. hongkongensis *(42.5%), *C. gigas *(42.3%) and *C. virginica *(43.0%).

**Table 6:** p-Distance (± Standard Error) of LURs repeats, subregions and motifs.

		pD	SE
Rep1	Rep2	0,004	0,001

A_1_	A_2_	0,000	0,000
A_1/2_	A''	0,362	0,032
A_1/2_	A'	0,449	0,035
A''	A'	0,505	0,033

B_1_	B_2_	0,002	0,001
B_2_	B	0,096	0,007
B_1_	B	0,098	0,007

C_1_	C_2_	0,010	0,005

**γ**_**C1**_	**γ**_**C**2_	0,008	0,005
**γ**_2_	**γ**_3_	0,012	0,006
**γ**_2_	**γ**_1_	0,015	0,007
**γ**_3_	**γ**_1_	0,019	0,009
**γ**_**C**1/**C**2_	**γ**_3_	0,346	0,027
**γ**_**C**1/**C**2_	**γ**_1/2_	0,350	0,027

Finally, in eight 2FD and seven 4FD codon families in females and in seven 2FD and seven 4FD codon families in males, the most frequently used codon does not match the tRNA anticodon. This has been observed in other metazoan mtDNA as well [46-50] and it suggests that strict codon-anticodon complementarity does not affect the codon composition of the genome. Deviations from equal frequency of the four nucleotides in 4FD sites are common in the animal mtDNA and have been attributed to several factors, such as unequal presence of the four nucleotides in the nucleotide pool, preference of the mitochondrial gamma DNA polymerase for specific nucleotides, or asymmetrical mutation rate owing to different duration of exposure of the lagging strand during replication [40,51-54].

Comparing the two *M. senhousia *sex linked genomes, the most conserved protein-coding genes are *cox1 *and *cob*, and the least conserved are *nad6 *and *atp8 *(Table 4). Synonymous (Ks) and non-synonymous (Ka) substitution values between the two genomes do vary (Table 4). Ka is particularly low for *cox1 *(0.042), whereas Ks is not (0.838), suggesting that this gene is under some selective constraint (Ka/Ks = 0.05). The conservation of *cox1 *is common in animal mtDNA [55,56]. In *cob *gene, both K values are lower than average (Table 4) with a Ka/Ks ratio's value (0.10) which is close to that of *cox1 *gene.

### The Large Unassigned Region (LUR)

**Figure 4:**
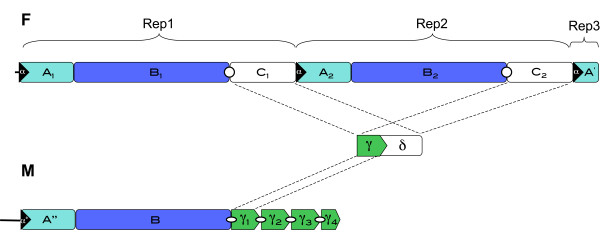
**Large Unassigned Regions (LURs)**. Schematic structure of female (F) and male (M) LURs in *Musculista senhousia*.

As mentioned, in the female genome the LUR (F-LUR) is 4,521 bp long and it is included between *trnE *and the 5'-end of *cox1 *gene (Figure 1 and 4, Table 1), while in the male it (M-LUR) is 2,844 bp long, and included between *trnN *and *trnE *genes (Figure 2 and 4, Table 2). Both start with a dissimilar sequence/spacer 20 and 237 bp long, respectively.

The F-LUR contains two large repeats (Figure 4: Rep1 and Rep2) about 2,150 bp long (2,149 Rep1; 2,151 Rep2), both subdividable in three regions: A, B and C (named A_1_, A_2_, B_1_, B_2_, C_1 _and C_2_; see Figure 4 and Additional File 3). Between Rep1 and Rep2, the A subregion is the most conserved (pD = 0.000, see Table 6) while C is the most variable, although with a low pD (0.010 ± 0.005). Overall, Rep1 and Rep2 have a pD of 0.004 ± 0.001. The region including the last 202 bp of the F-LUR shows some similarity (pD = 0.449 ± 0.035) to the A subregions (A_1 _and A_2_), for this reason it is indicated here as subregion A'.

**Figure 5:**
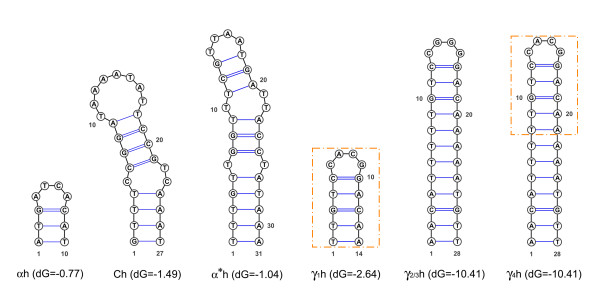
**LUR palindromes**. Sequences and structures of palindromic motifs located in the *Musculista senhousia *LURs.

All the A-type subregions (A_1_, A_2 _and A') start with a 46 bp conserved motif, named here α, that contains a 10 bp hairpin (αh; see Figure 5). Both the subunits C (C_1 _and C_2_) begin with a hairpin 27 bp long (Ch; Figure 5). The M-LUR contains an A-like subregion showing a pD of 0.362 ± 0.032 from A_1 _and A_2 _(Table 6), indicated as A'' (Figure 4). A'' starts with a 37 bp motif, here named α*, similar to α, but 9 bp shorter and with three mutations that allow the formation of a longer hairpin, here named α*h (31 bp; Figure 5), in comparison to the female hairpin αh. The M-LUR continues with the subunit B that is the most conserved region compared to the F-LUR showing a pD from B_1 _and B_2 _of 0.098 ± 0.007 and 0.096 ± 0.007 respectively (Table 6). At the 3' end of B there is a motif, indicated as γ (Figure 4) that is similar to the first part of the subunits C. γ is repeated four times in tandem. The length of γ_1_, γ_2 _and γ_3 _ranges from 268 and 265 bp while the last repeat, γ_4_, is truncated and measures 17 bp (Additional File 3; Figure 4). The pD among the γ motifs is low and ranges from 0.008 ± 0.005 in the female (between γ_c1 _and γ_c2_) and 0.019 ± 0.009 between γ_1 _and γ_3 _(Table 6). The pD of the γ motifs between male and female varies from 0.346 and 0.350 ± 0.027 (Table 6). At the 5' end of each γ motif a secondary structure is present (γ_1_h, γ_2_h, γ_3_h and γ_4_h respectively; Figure 5): γ_1_h is 14 bp long, while the other three are 28 bp long. γ_2_h and γ_3_h are identical, γ_4_h has a two bases mutation at the center of the loop and γ_1_h is identical to the upper portion of γ_4_h (see Figure 5).

Furthermore, in line with what has been found in other DUI bivalves, including *Mytilus*, an ORF coding for 121 amminoacids has been found in the F-LUR of *M. senhousia*. This protein was proposed to have a functional role in DUI. Detailed analyses on this novel DUI related putative protein have been published in a more comparative way (see [20]).

### The *cox2 *duplication in the male mtDNA

The male mtDNA contains an extra copy of the *cox2 *gene. This is not new for DUI animals, since the female mt genome of the marine clam *V. philippinarum *has a *cox2 *duplication as well (GenBank Acc. No. AB065375: Okazaki and Ueshima, unpublished).

In the female *Musculista*, the *cox2 *gene (*Fcox2*) is 660 bp long and is flanked by the "*cox1*/UR-6" and "UR-7/*atp8" *regions at the 5'- and 3'-end respectively (see Figure 1 and Table 1). In male mitochondrial genome, the two copies of *cox2 *are close to each other and linked by a little non coding region 41 bp long (UR-6). The two *cox2 *copies are located between "*cox1*/UR-5" and "UR-7/*atp8*" regions, and the first is 813 bp long, while the second is 690 bp long (Figure 2 and Table 2).

**Figure 6:**
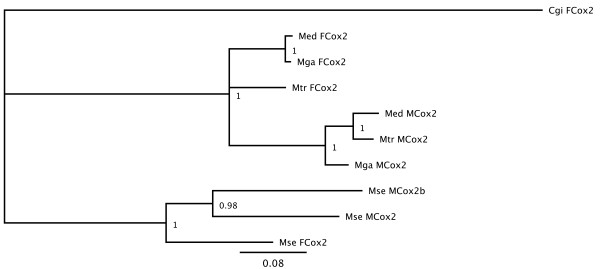
**Bayesian tree for the *cox2 *genes**. Cgi: *Crassostrea gigas*; Med: *Mytilus edulis*; Mga: *Mytilus galloprovincialis*; Mtr: *Mytilus trossulus*; Mse: *Musculista senhousia*.

Bayesian phylogenetic analyses on *Fcox2*, *Mcox2*(690 bp), *Mcox2*(813 bp) genes and their homologous in *Mytilus *species, demonstrated that *Fcox2 *is more closely related to the shorter *Mcox2 *(690 bp), rather than to the longer one (Figure 6). For this reason, the 813 bp long *Mcox2 *seems to be an extra copy of the gene, and thus it is referred here as *Mcox2b*.

## Discussion

### Gene content and order of F and M Mitochondrial genomes in *M. senhousia*

In *M. senhousia *both M and F mtDNAs share the same gene content and order, except for a duplicated *cox2 *gene in males, and include the typical gene content of bivalve mtDNA. It has to be noted, however, that a common feature of bivalves is the apparent lack of the *atp8 *gene. For instance, [2] mentioned that a lack of the *atp8 *gene is one of several unusual features of the *Mytilus *mt sequence. The *atp8 *gene was considered missing for almost all bivalve species studied so far, including *Crassostrea hongkongensis*, *C. gigas*, *C. virginica*, *Placopecten magellanicus*, *Argopecten irradians*, *Mizuhopecten yessoensis *and *Acanthocardia tuberculata*. On the contrary, the *apt8 *gene was found in *Hiatella arctica*, as well as in the female mitochondrial genome of the unionid bivalve *L. ornata *[28]. A remarkable observation is that *V. philippinarum*, another species with DUI [57], was recently found to contain a putative *atp8 *gene [58], which was not found in the first analyses; nonetheless, this gene apparently encodes 37 amino acids only and therefore has a questionable gene function. Finally, [23] examined ORFs from several bivalve mitochondrial genomes and found two novel ORFs (F-*orf-ur4 *and M-*orf-ur4*) in the largest unassigned region of F and M mytilid ones (UR-4: see [33]). BLASTN searches against EST_others (all ESTs except human and mouse) showed that both are transcribed in *Mytilus spp*. BLASTX and PSI-BLAST searches using inferred aminoacid sequences of F-*orf-ur4 *and M-*orf-ur4 *failed to detect any significant sequence similarity with known proteins, so the identity of those putative proteins is still unclear. Further analyses on structure and evolution patterns suggested that the novel ORFs "represent good candidates for the previously 'missing' *atp8 *in mytilid mtDNAs" [23]. Therefore, following [23], we also found *atp8 *putative genes in both sex-linked mitochondrial genomes of *M. senhousia*. Our *atp8 *genes share the same characteristics of the above mentioned proteins, so we are confident to annotate them as *Musculista atp8 *genes.

Generally speaking, most mtDNAs are characterized by strand asymmetry in term of gene distribution. In both *M. senhousia *mt genomes, all genes are transcribed from the same strand, i.e. the asymmetry is at its highest among Metazoa. Most marine bivalves also share this feature (*Mytilus *species-complex, *C. gigas*, *C. virginica*, *C. hongkongensis *and *V. philippinarum*). In contrast, this is not true for the two freshwater species *L. ornata *[28] and *Inversidens japanensis *(Acc. No. AB055625 and AB055624) (see also [59]). In other mollusks, a relatively small number of mitochondrial genes are transcribed from the second strand. The scaphopods *G. eborea *and *S. lobatum *are an exception, with about an equal number of genes encoded by each strand [31,58]. The occurrence of all genes in the same strand is a relatively rare phenomenon in metazoans and, in addition to bivalves, it has been reported in some annelids (*Lumbricus terrestris*, [60]*; Platynereis dumerilii*, [61]) and brachiopods (*Terebratulina retusa*, [62]; *Terebratalia transversa*, [42]; *Laqueus rubellus*, [63]). Actually, almost 10% of the mitochondrial genomes examined to date do have all genes encoded in the same strand [10]. Moreover, most of the above mentioned groups, including Bivalvia, are also characterized by strong differences in gene content and/or gene order. This allowed [10] to suggest a possible correlation between these two features.

The *trnS(AGN) *could not be located with tRNAscan-SE [64] because of the absence of the DHU arm and therefore of a normal cloverleaf structure (see [27] for a detailed discussion), so we used the ARWEN software [65] to identify it. This unconventional tRNA was found also in several other animal groups ([27] and references therein), and it evolved very early in Metazoa [66]. In vitro analyses confirmed its functionality [67].

In Table 7, the distribution of *trnS(UCN) *and *trnS(AGN) *among bivalves is reported (only complete mitochondrial genomes included; source: http://mi.caspur.it/mitozoa see [3]). Most of the species (22) have both the tRNAs, 7 only *trnS(UCN) *and 3 (including *M. senhousia*) only *trnS(AGN)*. *Placopecten magellanicus *have two copies of *trnS(UCN)*, while *Mizuhopecten yessoensis *seems to lack a Serine tRNA. [68] suggested that the secondary structure of a tRNA gene between a pair of protein genes is responsible for the precise cleavage of the polycistronic primary transcript. In the absence of a tRNA, this role can be played by a stem-loop structure, the 5'-end part of the gene itself, or a combination of the two. Potential hairpin structures at protein-protein gene junctions with no intervening tRNA have been reported in several studies (e.g., [6,33,39,69,70]). Our analysis demonstrated that putative hairpins are present in all the gene junctions in which a tRNA lacks, suggesting a functional role of such intergenic sequences (Figure 3).

**Table 7:** Serine tRNA [trnS(UCN) and trnS(AGN)] in bivalves.

Taxonomy	Species (GenBank Acc. No.)	Missing	UCN	AGN	UCN+AGN
***Pteriomorphia***					
Mytiloida; Mytiloidea; Mytilidae					
Crenellinae	*Musculista senhousia *(GU001953)			x	
Mytilinae	*Mytilus edulis *(AY823623)				x
	*Mytilus galloprovincialis *(AY363687)				x
	*Mytilus trossulus *(DQ198225)				x
Ostreoida; Ostreoidea; Ostreidae					
	*Saccostrea mordax *(FJ841968)			x	
	*Crassostrea angulata *(FJ841965)				x
	*Crassostrea ariakensis *(FJ841964)				x
	*Crassostrea gigas *(NC_001276)				x
	*Crassostrea hongkongensis *(EU266073)				x
	*Crassostrea iredalei *(FJ841967)				x
	*Crassostrea sikamea *(FJ841966)				x
Pectinoida; Pectinoidea; Pectinidae					
	*Mizuhopecten yessoensis *(FJ595959)	x			
	*Chlamys farreri *(EU715252)		x		
	*Mimachlamys nobilis *(FJ595958)		x		
	*Placopecten magellanicus *(NC_007234)*		xx		
	*Argopecten irradians *(NC_009687)				x
	*Argopecten irradians irradians *(DQ665851)			x	

***Heteroconchia***					
Myoida; Hiatelloidea; Hiatellidae					
	*Hiatella arctica *(NC_008451)				x
Veneroida; Cardioidea; Cardiidae					
	*Acanthocardia tuberculata *(NC_008452)				x
Veneroida; Lucinoidea; Lucinidae					
	*Loripes lacteus *(EF043341)				x
	*Lucinella divaricata *(EF043342)				x
Veneroida; Tellinoidea; Solecurtidae					
	*Sinonovacula constricta *(EU880278)		x		
Veneroida; Veneroidea; Veneridae					
	*Meretrix meretrix *(GQ463598)		x		
	*Meretrix petechialis *(EU145977)		x		
	*Venerupis philippinarum *(AB065374)		x		
	*Paphia euglypta *(GU269271)				x

***Palaeoheterodonta***					
Unionoida; Unionoidea; Unionidae					
	*Venustaconcha ellipsiformis *(FJ809752)				x
Ambleminae	*Quadrula quadrula *(FJ809750)				x
Anodontinae	*Cristaria plicata *(FJ986302)				x
Anodontinae	*Pyganodon grandis *(FJ809754)				x
Unioninae	*Hyriopsis cumingii *(FJ529186)				x
	*Inversidens japanensis *(AB055624)				x
	*Unio pictorium *(HM014131)				x

*: *Placopecten magellanicus *has two copies of *trnS(UCN)*

Note: only species with complete mitochondrial genomes available included.

### The Large Unassigned Region (LUR) and the sex-linked mt-DNA transmission

The structure of the F and M LUR palindromes found are reported on Figure 4 and 5. The presence of palindromes within a mtDNA CR is not new; in fact, the local fold symmetry created by the palindrome is thought to provide the site for DNA-binding proteins involved in the trascriptional machinery [71]. In more detail, palindromic motifs (and in general inverted repeats) have the potential to form single-stranded stem-loop cruciform structures which have been reported to be essential for replication of circular genomes in many prokaryotic and eukaryotic systems [72]. The redundancy of palindromic elements in the *Musculista *male LUR, when compared to that of the female, may be possibly related to an increased duplication ratio of the M mtDNA; we can also speculate that this feature may have some role in the process by which sperm mitochondrial DNA becomes dominant or exclusive of the male germline, although we know that this is also achieved through a differential segregation during early embryo development, and likely through a second, more strict, selection during primordial germ cells establishment (see [73]). Nevertheless, the question of how sperm mitochondrial DNA becomes dominant or the exclusive component of the male germline in DUI species still remains open, and may be the outcome of various coordinated processes.

### The duplication of the *cox2 *gene

One noteworthy finding of this analysis is the *cox2 *gene duplication in the male mtDNA, with the duplicated gene being longer than the original one, a feature that might be somehow related to DUI. In fact, an interesting analogy is evident with unionid bivalves, in which the male *cox2 *gene show a 200-codon extension, which is absent in the female mtDNA. Such a feature is found in all analyzed unionids so far, and it has been related to DUI functioning [21,22,74-76]. Actually, [21,22] proposed several hypotheses for the role the *cox2 *extension may have for DUI, but all are dependent upon identifying a specific function for it, which is not a trivial task. Moreover, they detected in the male gonad a poly-adenylated mRNA transcript of the *cox2 *gene that includes the extension, and they concluded that the extension is protein-coding and functional.

[21,22] also hypothesized that the COX2 protein extension might be involved in intracellular interactions determining the survival of the male mitochondrion. In other organisms, it has been shown that upon fertilization the sperm-derived mitochondria are targeted for elimination: a key process in sperm mitochondrial degradation is ubiquitination [77], in which mitochondria of paternal derivation are tagged with Ubiquitin and then degraded. In *Mytilus*, in which an Ubiquitin-like process has been proposed, this degradation would be sex-specific: the sperm-derived mitochondria survive in male embryos, whereas they are eliminated in females. All that considered, [21] proposed that the COX2 extension could be involved in blocking such elimination to ensure survival of the male mitochondrion, or, alternatively, the extension could play a role in the segregation of male mitochondria to the gonad. In either case, it should be possible to detect the protein product of the extension outside of the inner mitochondrial membrane. An *in situ *hybridization seemed to demonstrate that the unionid male COX2 is present on both inner and outer membranes of the sperm mitochondria (see Figure 4 in [74]).

According to the above mentioned rationales, we hypothesize that the duplicated *cox2b *gene in male *M. senhousia *may represent a variant of what found in unionoidean bivalves, with proper signals for DUI mitochondrial tagging lying in the COX2 protein extension of unionid bivalves, as well as in the duplicated COX2b protein of *Musculista*. A support to this view comes from the observation that an additional putative Trans Membrane Helix (TMH) is found in the 41 residue long tail of the *Musculista *COX2b, although this tail is considerably shorter that the unionid one (200 amminoacids). Actually, five putative TMHs were found in the unionid extended C-terminus of the male COX2, which led the Authors to hypothesize that it may have a functional significance for male unionoidean bivalve reproductive success [75,76].

In analogy, we suggest that COX2b might have some function related to mitochondrial tagging, like the COX2b and the Unionid COX2 extension. Further studies are needed to gain a more clear role of such proteins in the unusual DUI system of mitochondrial inheritance. Actually, a duplication similar to the *Musculista *one was also found in *V. philippinarum*, but quite surprisingly in the female mtDNA (see unpublished GenBank annotation). This suggests that *cox2 *duplication may be uncoupled with maleness. Moreover, no *Mytilus *genomes show a similar situation for *cox2 *or any other gene, so either duplicated genes or a *cox2 *tail may not be strictly necessary to sustain DUI.

## Conclusions

The characteristics of the *Musculista *sex-linked mtDNAs evidently add to the knowledge of DUI systems, and highlight some unexpected features, shared among distantly related DUI species. Since it is commonly accepted that DUI is rather a variation of Strict Maternal Inheritance, than a completely different mechanism, we think that DUI is a good experimental model to better understand the general rules, as well as the molecular features of Metazoan mitochondrial inheritance (see [18], for a detailed discussion). For the above mentioned reasons, the complete mtDNA genome characterization of DUI bivalves is not only a mere descriptive exercise, but rather a first step to unravel the complex genetic signals allowing Doubly Uniparental Inheritance of mitochondrial DNA, and the evolutionary implications of such unusual transmission route in mitochondrial genome evolution in Bivalvia.

## Methods

### Sample Collection

Alive *M. senhousia *specimens from Venice Lagoon (Italy) were used for this analysis. Males and females were stimulated to spawn gametes in seawater supplemented with hydrogen peroxide, according to [78]. Each emission was analyzed with a light microscope to sex specimens. A total of 10 sperm and 10 egg samples were then collected after a gentle centrifugation (3,000 g). Seawater was removed, and ethanol added before storing samples at -20°C.

### PCR analyses

Total genomic DNA was extracted using the DNeasy Tissue Kit (Qiagen), and partial sequences of cytochrome b (*cob*) and mitochondrial ribosomal large subunit RNA (*rrnL*) were amplified and directly sequenced (primers reported in Table 8), as described in [79]. Sequencing reactions were performed on both strands with BigDye Terminator Cycle Sequencing Kit according to supplier's instructions (Applied Biosystem) in a 310 Genetic Analyzer (ABI) automatic sequencer.

**Table 8:** Primer sequences.

Primer name	Sequence
cobR^1^	5'-GCRTAWGCRAAWARRAARTAYCAYTCWGG-3'
cobF^1^ 16Sbr^2^	5'-GGWTAYGTWYTWCCWTGRGGWCARAT-3' 5'-CCGGTCTGAACTCAGATCACGT-3'
16Sar^2^	5'-CGCCTGTTTATCAAAAACAT-3'
F-cob383R F-16S142F	5'-TAGGAGTTTTTATAGGGTCTGC-3' 5'-ACCTGAAGTTGTCTCATTTACC-3'
M-cob386R M-16S103F	5'-GGATAGGAGTTTTTATAGGGTCTGC-3' 5'-GTGAATTTCTTAGAGTGACGATTA-3'

^1 ^J.L. Boore, personal communication; ^2 ^[88]

The 20 sequences obtained for both F and M genomes were aligned (not shown), and, after checking for variable sites, used to design sex-specific primers to amplify the entire mitochondrial genome in two overlapping fragments by long PCR reactions. LongPCR was performed on one *Musculista *specimen per sex. To obtain the F genome, F-cob383R and F-16S142F primers were used. The M genome was amplified with M-cob386R and M-16S103F. Both pairs of primers amplified a fragment of 10-11 kb respectively. Long PCR primer sequences are reported in Table 1. LongPCR amplifications were performed on a Gene Amp^® ^PCR System 2720 (Applied Biosystem) in 50 μl reaction volume composed of 31.5 μl of sterilized distilled water, 10 μl of 5 × Herculase II Fusion Reaction Buffer, 0.5 μl of dNTPs mix (25 mM each dNTP), 1.25 μl of each primer (10 μM), 5 μl of DNA template (25-50 ng) and 0.5 μl of Herculase II Fusion DNA Polymerase. Reaction conditions were according to supplier's recommendations: initial denaturation at 95°C for 5 min and then incubated at 95°C for 20 s, 50°C for 20 s, and 68°C for 10 min for 30 cycles and 68°C for 8 min for a final extension. Long-PCR fragments were then purified using Wizard^® ^SV Gel and PCR Clean-Up System (Promega).

### Shotgun cloning

Sequencing of the two LongPCR fragments was done using shotgun cloning: amplicons were randomly sheared to 1.2-1.5 kb DNA segments using a HydroShear device (GeneMachines). Sheared DNA was blunt end repaired at room temperature for 60 min using 6 U of T4 DNA Polymerase (Roche), 30 U of DNA Polymerase I Klenow (NEB), 10 μl of dNTPs mix, 13 μl of 10 × NEB buffer 2 in a 115 μl total volume, and then gel purified using the Wizard^® ^SV Gel and PCR Clean-Up System (Promega). The resulting fragments were ligated into the SmaI site of a pUC18 cloning vector using the Fast-Link DNA ligation Kit (Epicentre) and electroporated into One Shot^® ^TOP10 Electrocomp™ *Escherichia coli *cells (Invitrogen) using standard protocols. Clones were screened by PCR using M13 universal primers and recombinants were purified using Multiscreen (Millipore) according to the manufacturer's instructions. Clones were sequenced using M13 universal primers by Macrogen Inc. (Korea).

Raw sequences were manually corrected, and then assembled into contigs with Sequencher v.4.6 (Gene Codes). Hence, the final assemblies were based on a minimum sequence coverage of 3×.

### Secondary structures and annotation

The tRNA genes were identified by their secondary structure using ARWEN [65], with invertebrate mitochondrial codon predictors. Analysis of Open Reading Frames (ORFs) was performed with the ORF Finder program of NCBI http://www.ncbi.nlm.nih.gov/projects/gorf/ using the invertebrate mitochondrial genetic code. Sequences were identified using BLASTX, PSI-BLAST [80] and BLASTN [81] as implemented by the NCBI website http://www.ncbi.nlm.nih.gov/.

For all protein coding genes, alignments were computed with ClustalW [82].

When analyzing sequence variability, pairwise p-Distances (pD), their mean values and standard errors (by the bootstrap procedure) were computed with MEGA v.5.03 [83]. In order to avoid any model of DNA substitution that can affect statistics (see [79]), the use of a pD was preferred.

The divergence of protein genes in synonymous (Ks) and non-synonymous (Ka) sites was calculated by the modified Nei-Gojobori method with the Jukes-Cantor correction; the pD at the residue level was also calculated within the MEGA v.5.03 environment [83].

Two-fold, and four-fold degenerated positions were identified using DnaSP v.5 [84]. The Sequence Manipulation Suite (http://www.bioinformatics.org/sms2; [85]) was used to estimate codon usage. Potential DNA secondary structures near or at the 5'-end of protein genes were predicted using the UNAFold software package [86] available on the DINAMelt web server (http://mfold.rna.albany.edu/?q=DINAMelt; [86]).

Bayesian analyses on *cox2 *genes was performed with the MrBayes 3.1 (5,000,000 generations; [87]).

## Authors' contributions

MP conceived the study, participated in its design and coordination and drafted the manuscript. AR carried out the lab work and performed part of the analysis. LM and FG performed part of the analysis and drafted the manuscript. All authors read and approved the final manuscript.

## Supplementary Material

Additional file 1 **The Unassigned Regions (URs) in the female and male mtDNAs of *Musculista senhousia***. Annotation and length of Unassigned Regions (URs) in the female (Mse_URs_F) and male (Mse_URs_M) mtDNAs of *Musculista senhousia*. Click here for file

Additional file 2 **tRNAs in the female and male mtDNAs of *Musculista senhousia***. Annotation, length and structures of tRNAs in the female (Mse_trn_F) and male (Mse_trn_M) mtDNAs of *Musculista senhousia*. Click here for file

Additional file 3 **Structure of the female (F-LUR) and male (M-LUR) Large Unassigned Regions of *Musculista senhousia *mtDNA**. Schematic table of repeats and hairpin structures in the Large Unassigned Regions (LURs) of the female and male *Musculista senhousia *mtDNAs (F-LUR and M-LUR). Click here for file
