# Supplementary material for: Mitochondrial genomes and Doubly Uniparental Inheritance: new insights from Musculista senhousia sex-linked mitochondrial DNAs (Bivalvia Mytilidae)
Source: BMC Genomics. 2011 Sep 6;12:442. doi: 10.1186/1471-2164-12-442 (PMC3176263; doi:10.1186/1471-2164-12-442)
Supplement: Additional file 1 — The Unassigned Regions (URs) in the female and male mtDNAs of Musculista senhousia. Annotation and length of Unassigned Regions (URs) in the female (Mse_URs_F) and male (Mse_URs_M) mtDNAs of Musculista senhousia. [file 1471-2164-12-442-S1.PDF]

Mse_URs_F

Name	Starts	Stops	Length
UR-1	391	625	235
UR-2	692	1234	543
UR-3	1300	1315	16
UR-4	1382	1391	10
UR-5	1458	1564	107
LUR	1632	6152	4521
UR-6	7737	8114	378
UR-7	8775	8832	58
UR-8	8968	9051	84
UR-9	9766	9791	26
UR-10	11032	11049	18
UR-11	11115	11123	9
UR-12	11249	11268	20
UR-13	12779	12828	50
UR-14	13774	13855	82
UR-15	14711	14721	11
UR-16	14793	14797	5
UR-17	14866	14878	13
UR-18	14946	14977	32
UR-19	15043	15047	5
UR-20	15115	15159	45
UR-21	15224	15259	36
UR-22	16253	16385	133
UR-23	16449	16486	38
UR-24	16551	16695	145
UR-25	16912	16988	77
UR-26	18805	18843	39
UR-27	20164	20213	50
UR-28	20281	20285	5
UR-29	20354	20360	7
TOTAL			6798

Mse_URs_M

Name	Starts	Stops	Length
UR-1	376	433	58
UR-2	502	533	32
UR-3	600	618	19
LUR	754	3597	2844
UR-4	3669	3708	40
UR-5	5293	5852	560
UR-6	6666	6706	41
UR-7	7397	7402	6
UR-8	7595	7612	18
UR-9	8327	8347	21
UR-10	9589	9606	18
UR-11	9672	9681	10
UR-12	9807	9825	19
UR-13	11418	11472	55
UR-14	12418	12444	27
UR-15	13367	13377	11
UR-16	13446	13464	19
UR-17	13529	13554	26
UR-18	13622	13625	4
UR-19	13697	13737	41
UR-20	13805	13840	36
UR-21	14900	14985	86
UR-22	15050	15183	134
UR-23	15400	15464	65
UR-24	17295	17338	44
UR-25	18668	18710	43
UR-26	18778	18781	4
UR-27	18849	18863	15
TOTAL			4296
