# Supplementary material for: Mitochondrial genomes and Doubly Uniparental Inheritance: new insights from Musculista senhousia sex-linked mitochondrial DNAs (Bivalvia Mytilidae)
Source: BMC Genomics. 2011 Sep 6;12:442. doi: 10.1186/1471-2164-12-442 (PMC3176263; doi:10.1186/1471-2164-12-442)
Supplement: Additional file 2 — tRNAs in the female and male mtDNAs of Musculista senhousia. Annotation, length and structures of tRNAs in the female (Mse_trn_F) and male (Mse_trn_M) mtDNAs of Musculista senhousia. [file 1471-2164-12-442-S2.PDF]

Mse_trn_F

Name	Starts	Stops	Length	Strand
trnY	626	691	66	H
trnH	1235	1299	65	H
trnI	1316	1381	66	H
trnN	1392	1457	66	H
trnE	1565	1631	67	H
trnT	9792	9858	67	H
trnD	11050	11114	65	H
trnR	11124	11189	66	H
trnS(AGN)	11191	11248	58	H
trnG	11269	11336	68	H
trnK	14722	14792	71	H
trnF	14798	14865	68	H
trnP	14879	14945	67	H
trnL(CUN)	14978	15042	65	H
trnC	15048	15114	67	H
trnL(UUR)	15160	15223	64	H
trnM(AUA)	16386	16448	63	H
trnV	16487	16550	64	H
trnA	18739	18804	66	H
trnW	20214	20280	67	H
trnQ	20286	20353	68	H
trnM(AUG)	20361	20427	67	H

Female tRNAs of *Musculista senhousia*

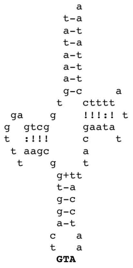

mtRNA-Tyr(gta)
66 bases, %GC = 31.8
Sequence [626,691]

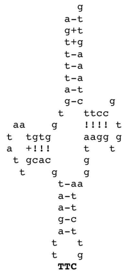

mtRNA-Glu(ttc)
67 bases, %GC = 35.8
Sequence [1565,1631]

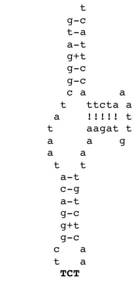

D-loop mtRNA-(Ser)(tct)
58 bases, %GC = 34.5
Sequence [11191,11248]

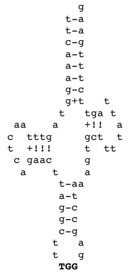

mtRNA-Pro(tgg)
67 bases, %GC = 35.8
Sequence [14879,14945]

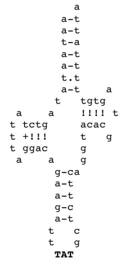

mtRNA-(Met)(tat)
63 bases, %GC = 28.6
Sequence [16386,16448]

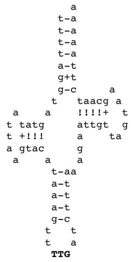

mtRNA-Gln(ttg)
68 bases, %GC = 20.6
Sequence [20286,20353]

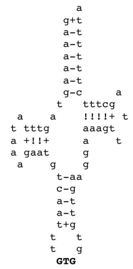

mtRNA-His(gtg)
65 bases, %GC = 26.2
Sequence [1235,1299]

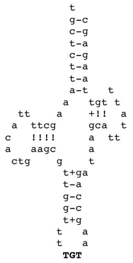

mtRNA-Thr(tgt)
67 bases, %GC = 35.8
Sequence [9792,9858]

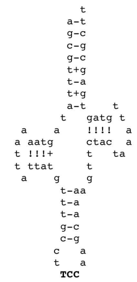

mtRNA-Gly(tcc)
68 bases, %GC = 32.4
Sequence [11269,11336]

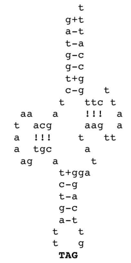

mtRNA-Leu(tag)
65 bases, %GC = 35.4
Sequence [14978,15042]

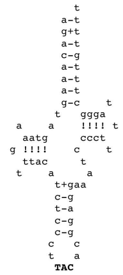

mtRNA-Val(tac)
64 bases, %GC = 39.1
Sequence [16487,16550]

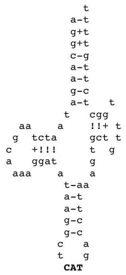

mtRNA-Met(cat)
67 bases, %GC = 37.3
Sequence [20361,20427]

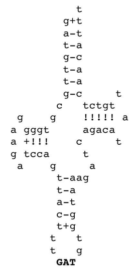

mtRNA-Ile(gat)
66 bases, %GC = 39.4
Sequence [1316,1381]

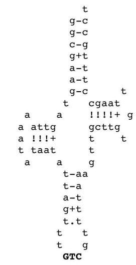

mtRNA-Asp(gtc)
65 bases, %GC = 32.3
Sequence [11050,11114]

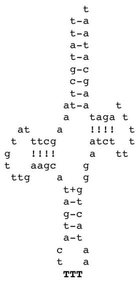

mtRNA-(Lys)(ttt)
71 bases, %GC = 25.4
Sequence [14722,14792]

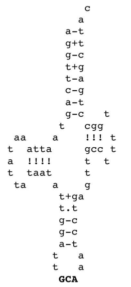

mtRNA-Cys(gca)
67 bases, %GC = 34.3
Sequence [15048,15114]

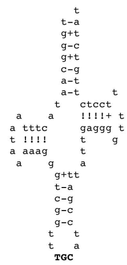

mtRNA-Ala(tgc)
66 bases, %GC = 39.4
Sequence [18739,18804]

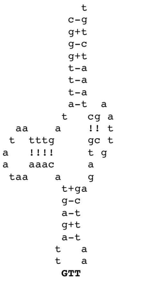

mtRNA-Asn(gtt)
66 bases, %GC = 28.8
Sequence [1392,1457]

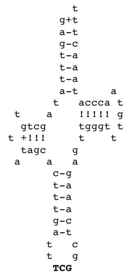

mtRNA-Arg(tcg)
66 bases, %GC = 36.4
Sequence [11124,11189]

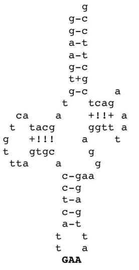

mtRNA-Phe(gaa)
68 bases, %GC = 44.1
Sequence [14798,14865]

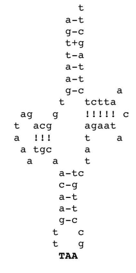

mtRNA-Leu(taa)
64 bases, %GC = 32.8
Sequence [15160,15223]

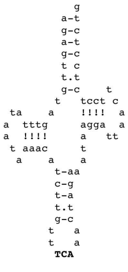

mtRNA-(Trp)(tca)
67 bases, %GC = 29.9
Sequence [20214,20280]

Mse_trn_M

Name	Starts	Stops	Length	Strand
trnY	434	501	68	H
trnH	534	599	66	H
trnI	619	688	70	H
trnN	687	753	67	H
trnE	3598	3668	71	H
trnT	8348	8416	69	H
trnD	9607	9671	65	H
trnR	9682	9745	64	H
trnS(AGN)	9747	9806	60	H
trnG	9826	9893	68	H
trnK	13299	13366	68	H
trnF	13378	13445	68	H
trnP	13465	13528	64	H
trnL(CUN)	13555	13621	67	H
trnC	13626	13696	71	H
trnL(UUR)	13738	13804	67	H
trnM(AUA)	14835	14899	65	H
trnV	14986	15049	64	H
trnA	17230	17294	65	H
trnW	18711	18777	67	H
trnQ	18782	18848	67	H
trnM(AUG)	18864	18930	67	H

Male tRNAs of *Musculista senhousia*

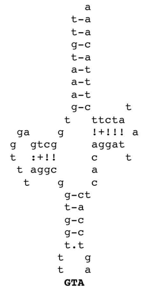

mtRNA-Tyr(gta)
68 bases, %GC = 39.7
Sequence [434,501]

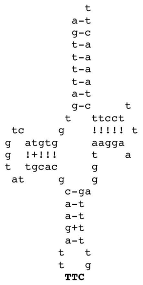

mtRNA-Glu(ttc)
71 bases, %GC = 35.2
Sequence [3598,3668]

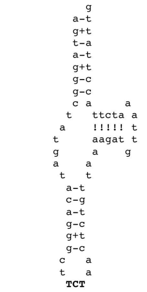

mtRNA-(Ser)(tct)
60 bases, %GC = 35.0
Sequence [9747,9806]

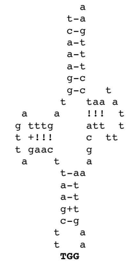

mtRNA-Pro(tgg)
64 bases, %GC = 26.6
Sequence [13465,13528]

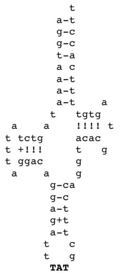

mtRNA-(Met)(tat)
65 bases, %GC = 36.9
Sequence [14835,14899]

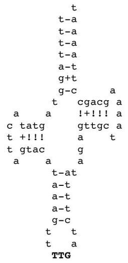

mtRNA-Gln(ttg)
67 bases, %GC = 26.9
Sequence [18782,18848]

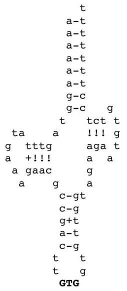

mtRNA-His(gtg)
66 bases, %GC = 36.4
Sequence [534,599]

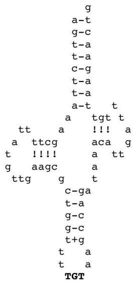

mtRNA-Thr(tgt)
69 bases, %GC = 33.3
Sequence [8348,8416]

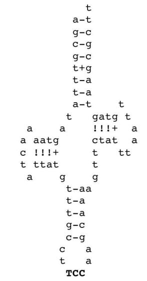

mtRNA-Gly(tcc)
68 bases, %GC = 30.9
Sequence [9826,9893]

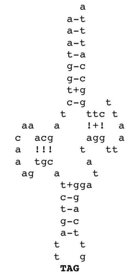

mtRNA-Leu(tag)
67 bases, %GC = 35.8
Sequence [13555,13621]

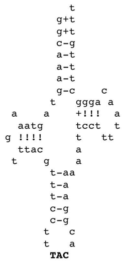

mtRNA-Val(tac)
64 bases, %GC = 34.4
Sequence [14986,15049]

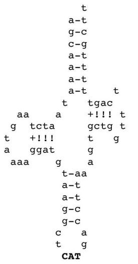

mtRNA-Met(cat)
67 bases, %GC = 34.3
Sequence [18864,18930]

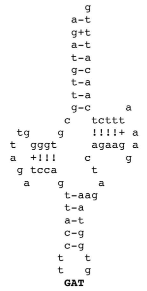

mtRNA-Ile(gat)
70 bases, %GC = 40.0
Sequence [619,688]

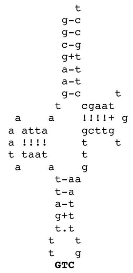

mtRNA-Asp(gtc)
65 bases, %GC = 30.8
Sequence [9607,9671]

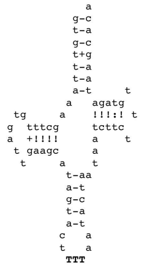

mtRNA-(Lys)(ttt)
68 bases, %GC = 27.9
Sequence [13299,13366]

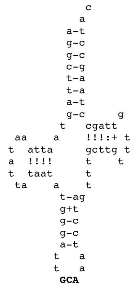

mtRNA-Cys(gca)
71 bases, %GC = 32.4
Sequence [13626,13696]

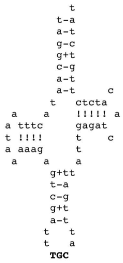

mtRNA-Ala(tgc)
65 bases, %GC = 29.2
Sequence [17230,17294]

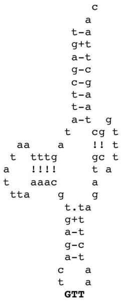

mtRNA-Asn(gtt)
67 bases, %GC = 29.9
Sequence [687,753]

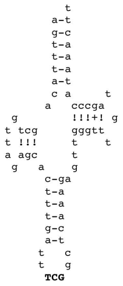

mtRNA-Arg(tcg)
64 bases, %GC = 40.6
Sequence [9682,9745]

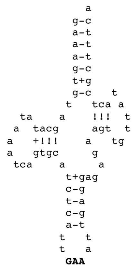

mtRNA-Phe(gaa)
68 bases, %GC = 35.3
Sequence [13378,13445]

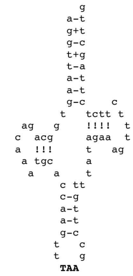

mtRNA-Leu(taa)
67 bases, %GC = 37.3
Sequence [13738,13804]

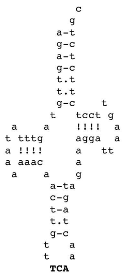

mtRNA-(Trp)(tca)
67 bases, %GC = 31.3
Sequence [18711,18777]
