# Supplementary material for: Mitochondrial genomes and Doubly Uniparental Inheritance: new insights from Musculista senhousia sex-linked mitochondrial DNAs (Bivalvia Mytilidae)
Source: BMC Genomics. 2011 Sep 6;12:442. doi: 10.1186/1471-2164-12-442 (PMC3176263; doi:10.1186/1471-2164-12-442)
Supplement: Additional file 3 — Structure of the female (F-LUR) and male (M-LUR) Large Unassigned Regions of Musculista senhousia mtDNA. Schematic table of repeats and hairpin structures in the Large Unassigned Regions (LURs) of the female and male Musculista senhousia mtDNAs (F-LUR and M-LUR). [file 1471-2164-12-442-S3.PDF]

F-LUR

Rep. Region	Subunit	Length	Motif	Length	Hairpin	Length
A	A_1	308	α	46	αh	10
	A_2	308	α	46	αh	10
	A'	202	α	46	αh	10
B	B_1	1429	-	-	-	-
	B_2	1430	-	-	-	-
C	C_1	412	γ_{C1}	265	Ch	27
			δ_{C1}	147	-	-
	C_2	413	γ_{C2}	266	Ch	27
			δ_{C2}	147	-	-

M-LUR

Rep. Region	Subunit	Length	Motif	Length	Hairpin	Length
-	A"	318	α*	37	α*h	31
-	B	1433	-	-	-	-
-	-	-	γ_1	268	γ_1h	14
			γ_2	265	γ_2h	28
			γ_3	267	γ_3h	28
			γ_4	17	γ_4h	28
